# Enhancing Bioavailability and Stability of Curcumin Using Solid Lipid Nanoparticles (CLEN): A Covenant for Its Effectiveness

**DOI:** 10.3389/fbioe.2020.00879

**Published:** 2020-10-15

**Authors:** Tanvi Gupta, Joga Singh, Sandeep Kaur, Simarjot Sandhu, Gurpal Singh, Indu Pal Kaur

**Affiliations:** University Institute of Pharmaceutical Sciences, Panjab University, Chandigarh, India

**Keywords:** oral bioavailability, enhanced solubility, improved stability, pharmacokinetics, photodegradation

## Abstract

Curcumin, very rightly referred to as “a wonder drug” is proven to be efficacious in a variety of inflammatory disorders including cancers. Antiaging, anti-inflammatory, antioxidant, antitumor, chemosensitizing, P-gp efflux inhibiting, and antiproliferative activity are some of the striking features of curcumin, highlighting its importance in chemotherapy. Curcumin inhibits Bcl-2, Bcl-XL, VEGF, c-My*c*, ICAM-1, EGFR, STAT3 phosphorylation, and cyclin D1 genes involved in the various stages of breast, prostate, and gastric cancer proliferation, angiogenesis, invasion, and metastasis. The full therapeutic potential of curcumin however remains under explored mainly due to poor absorption, rapid metabolism and systemic elimination culminating in its poor bioavailability. Furthermore, curcumin is insoluble, unstable at various pH and is also prone to undergo photodegradation. Nanotechnology can help improve the therapeutic potential of drug molecules with compromised biopharmaceutical profiles. Solid lipid nanoparticles (SLNs) are the latest offshoot of nanomedicine with proven advantages of high drug payload, longer shelf life, biocompatibility and biodegradability, and industrial amenability of the production process. We successfully developed CLEN (Curcumin encapsulated lipidic nanoconstructs) containing 15 mg curcumin per ml of the SLN dispersion with highest (till date, to our knowledge) increase in solubility of curcumin in an aqueous system by 1.4 × 10^6^ times as compared to its intrinsic solubility of 11 ng/ml and high drug loading (15% w/v with respect to lipid matrix). Zero-order release kinetics observed for CLEN versus first order release for free curcumin establish controlled release nature of the developed CLEN. It showed 69.78 times higher oral bioavailability with respect to free curcumin; 9.00 times higher than a bioavailable marketed formulation (CurcuWIN^®^). The formulation showed 104, 13.3, and 10-times enhanced stability at pH 6.8, 1.2, and 7.4, respectively. All these factors ensure the efficacy of CLEN in treating cancer and other inflammatory diseases.

## Introduction

Phytochemicals are biologically active compounds that are synthesized within the flora to produce a characteristic taste, color, and aroma and are beneficial to humans when taken in appropriate amounts. Around 20,000 plants recorded in Ayurveda are used for their medicinal value in treating a variety of ailments. Modern medicine is starting to recognize the beneficial effects of herbs for therapy, including curcumin, which is extensively studied, as evidenced by the publication of more than 6000 research publications appearing on curcumin in less than 20 years (Prasad et al., 2014).

Curcumin is the principal active ingredient of *Curcuma longa* (*Linn.*), popularly known as turmeric [Zingiberaceae family] (Gupta et al., 2012; Rahmani et al., 2018). It is native to the Indian subcontinent and Southeast Asia and is very popular as a condiment in the cuisines of this continent. Researchers across the globe have explored its antimicrobial, antioxidant, antidiabetic, anti-inflammatory, and anti-tumor capabilities (Aggarwal and Harikumar, 2009). Curcumin is documented for use in gastrointestinal problems (through downregulation of NF-kB, IL-6, TRPV-1, and STAT3) (Rajasekaran, 2011; Lopresti, 2018), liver diseases (inhibition of TGF-β) (Espinoza and Muriel, 2009), inflammatory conditions (suppression of TNF-α, IL-6, 8, and 12, COX-2, and iNOS) (Fadus et al., 2017; Hewlings and Kalman, 2017), and as an anticancer drug (repressing IL-10 and 18, AP-1, Pgp {Akt/IKKα- β/NF-kB axis} and activation of MAPK) (Liu and Ho, 2018; Tomeh et al., 2019). Curcumin shows potential in cancer management by significantly decreasing the elevated levels of Cyclin D1 and CDK4 (proteins involved in cell cycle progression) and thus arresting cell cycle at the G1 phase, causing cell growth inhibition/apoptosis (Vutakuri, 2018; Tan and Norhaizan, 2019). Curcumin improves reduced glutathione (GSH) levels coupled with the downregulation of NF-kB, both of which are useful for cancer control.

Adjuvant therapy involving the use of curcumin with conventional anticancer drugs, including doxorubicin, paclitaxel, cisplatin, etoposide, 5-fluorouracil, docetaxel, mitomycin C, tamoxifen, and cyclophosphamide is reported to enhance the chemotherapeutic effect of the latter. Curcumin acts by reducing transcriptional activation of the Pgp promoter (p65/p50) and inhibition of the CYP enzymes (CYP3A4, CYP2D6) (Saghatelyana et al., 2020) that are involved in drug metabolism. Curcumin also reduced cancer metastasis and suppressed expression of MMP-9, NF-kB, and COX-2 when administered in combination with other chemotherapeutic agents (Wilken et al., 2011; Wang et al., 2016).

Curcumin is often referred to as a ‘wonder drug’ because of the multiplicity of effects displayed by it, which are especially useful for management of multitarget diseases like cancers. However, the wide spectrum of curcumin remains under-exploited due to its insolubility (aqueous solubility of about 11 ng/ml), rapid metabolism, poor bioavailability, and physiological instability (undergoes degradation in water and at various physiological pH conditions), limiting its clinical application (Liu et al., 2016). Therefore, there is a need to develop a robust technology that can overcome the biopharmaceutical flaws inherent in the curcumin molecule (Flora et al., 2013).

Recent reports have proved that nanotechnology can be used to manage various issues with curcumin bioavailability (Gera et al., 2017; Karthikeyan et al., 2020). Basniwal et al. (2014) prepared nanoparticles of curcumin (2–40 nm) using a wet milling technique and carried out *in-vitro* testing against A549, HepG2, and A431 cancer cell lines (representing lung, liver, and skin cancer), which proved to have a significant antiproliferative effect in comparison to free curcumin. A nanogel containing curcumin exhibited almost three times potency in comparison with plain curcumin (Khosropanaha et al., 2016) in MDA-MB231 cells (breast cancer). Furthermore, curcumin nanoparticles exhibited significantly more uptake in both PC3 and HEK cell lines (prostate cancer cell lines and human embryonic kidney cell line). Specifically, the nanoparticles of curcumin have shown more toxic behavior in PC3. A study documented that the viability with nanocurcumin was lower at all the concentrations in comparison to curcumin in both the cell lines (Adahouna et al., 2017). In another investigation, curcumin nanoparticles showed 19 fold more growth inhibition on Colon-26 (colorectal cancer cell line) cells in contrast to free curcumin. This phenomenon was seen mainly due to greater binding and enhanced cellular uptake of nanoparticles (Chaurasia et al., 2016). Sustained release of curcumin from silk fibroin nanoparticles improved its cellular uptake into the cancer cells (HCT116; colon cancer cell) and reduced its cytotoxic effect on normal healthy cells (Xie et al., 2017).

Solid lipid nanoparticles (SLNs) are the latest development in the field of nanotechnology, offering the desirable properties of a high drug pay load, such as biocompatibility, small size, protection against chemical degradation, physical stability, enhanced cellular uptake, and controlled release in comparison to other nano delivery vehicles, including liposomes, nanoemulsions, micelles, and polymeric nanoparticles (Sun et al., 2012; Flora et al., 2013).

## Materials and Methods

### Chemicals and Materials

The curcumin was donated by Sunpure Extracts Pvt. Ltd., New Delhi, India. Compritol^®^ 888 ATO, Glyceryl Monostearate (GMS) was gifted by Gattefosse India Pvt. Ltd., and phospholipon 90G was also gifted by Lipoid, GmbH Germany. β-glucuronidase was purchased from Megazyme Ltd., Ireland. Tween 80 and polyethylene glycol 600 (CDH, New Delhi, India) were purchased from local vendors. CurcuWIN^®^ was purchased as an online product from OmniActive Health Technologies. Acetonitrile (ACN), chloroform, and methanol (HPLC grade) manufactured by Merck Schuchardt OHG, Hohenbrunn, Germany, were also purchased locally. HPLC grade water was produced by a Milli-DI system by Millipore (Billerica, MA, United States). Syringe filters were purchased from Waters India Pvt. Ltd. All other reagents used in the study were of analytical grade.

### Preparation of Curcumin Loaded Solid Lipid Nanoparticles (CLEN)

**FIGURE 1:**
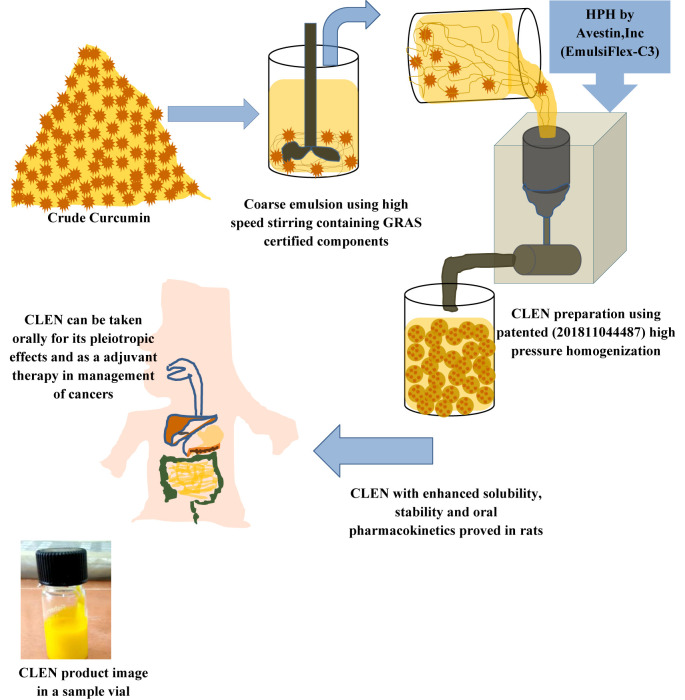
Diagrammatic representation of CLEN preparation.

CLEN were prepared using a high pressure hot homogenization technique. The aqueous phase was prepared by adding tween 80, phospholipon 90G, and water in a beaker and heated to around 80°C. Lipid [Compritol^®^888 ATO and GMS (4:1)] was melted at 70–75°C and curcumin, dissolved in polyethylene glycol 600 was added to it. The obtained lipid mix was added to the aqueous phase under high-speed homogenization (8000 rpm for 8 min) to obtain a coarse emulsion. The latter was passed through a high pressure homogenizer (3 cycles; 500 psi) and the SLNs were formed by cooling the obtained dispersion to room temperature (Figure 1). A graphical representation of the process and its proposed applications is depicted in Figure 1.

Various formulations were prepared by varying types and concentrations of the lipid and by the concentration of phospholipon 90G to achieve a 1.5% w/v curcumin in the SLN dispersion.

### Characterization of CLEN

#### Assay [Total Drug Content (TDC)] of Curcumin in CLEN Dispersion

Depending upon the solubility of Compritol^®^888 ATO, and GMS, methanol: chloroform (1:1) was chosen as a solvent for disrupting the SLNs. 1 ml of CLEN was suitably diluted to 5000 times with chloroform: methanol solvent system. The obtained solution was analyzed spectrophotometrically at λmax 425 nm using the corresponding blank. TDC was determined using the following equation:

$$\text{Total}\text{drug}\text{conten}t\left(\%\right)=\frac{\text{observed}\text{drug}\text{content}}{\text{Theoretical}\text{drug}\text{content}}\times 100$$

#### Determination of Entrapment Efficiency (EE)

The EE of CLEN was determined using the dialysis membrane method. Membrane (cut off 7 kDa MW) was soaked in double distilled water overnight before use. 1 mL of CLEN dispersion was placed in the pre-soaked dialysis bag tied at both ends and dialyzed against methanol (100 mL) at room temperature for 45 min. The amount of the drug remaining in the dialysis bag was analyzed spectrophotometrically following appropriate dilution (5000 times) with methanol: chloroform (1:1) to calculate the amount of drug entrapped within the SLNs. EE was determined using the following equation:

$$\text{Entrapment}\mathrm{Efficiency}\left(\%\right)=\frac{\text{Entrapped}\text{drug}}{\text{Total}\text{drug}\text{content}}\times 100$$

#### Particle Size Analysis, Polydispersity Index (PDI) and Zeta Potential

The mean diameter, PDI, and zeta potential of CLEN (*n* = 6) were determined after appropriate dilution (20 times) with double distilled water using Delsa^TM^ Nano C Particle Analyzer (Beckman Coulter, United States).

#### Field Emission Scanning Electron Microscopy (FESEM)

CLEN were observed microscopically using FESEM (H-7500, Hitachi Ltd., Japan) for uniformity of size, shape, and physical stability characteristic, i.e., aggregation or irregularity. FESEM has narrow probing beams at low and high electron energy which provides improved spatial resolution while minimizing sample damage. It provides topographical information at magnifications of 250-1,000,000X with ion-free images. A drop of the sample was placed on the carbon-coated copper grid to form a thin film on the grid. The grid was air dried and samples were viewed under FESEM.

#### Powder X-Ray Diffraction (PXRD)

The crystalline or amorphous nature of SLNs was confirmed by X-ray diffraction measurements carried out by an X-ray diffractometer. PXRD studies were performed by exposing the samples to Cu K_α_ radiation (45 kV, 40 mA) and scanning from 5° to 50°, 2θ at a step size of 0.017° and scan step time of 25 s. The instrument measures interlayer spacing d which is calculated from the scattering angle θ, using Bragg’s equation nλ = 2d sinθ where λ is the wavelength of the incident X-ray beam and n is the order of the interference. Obtained XRD patterns were compared for characteristic drug peak intensity.

#### Differential Scanning Calorimeter (DSC)

Differential scanning calorimeter thermograms of curcumin, CLEN, blank SLNs, and lipid mixture [Compritol^®^888 ATO and GMS (4:1)] were recorded on a Q20 differential scanning calorimeter. Samples were placed in aluminum hermetic pans and heated at a predefined rate of 10°C/min over the temperature range from 30 to 300°C in the nitrogen atmosphere.

#### Fourier Transform Infra-Red (FTIR)

Fourier transform infra-red spectra of curcumin, CLEN, blank SLNs, and lipid mixture [Compritol^®^888 ATO and GMS (4:1)] were recorded using the KBr pellet technique using 60 MHz Varian EM 360 (PerkinElmer, United States). The peaks obtained were compared for any significant changes.

### *In vitro* Release of CLEN

The drug release studies were performed using a mixture of double distilled water and methanol (1:1, v/v) as the dissolution medium and using the dialysis bag method. The dialysis bags were soaked in double distilled water for 12 h prior to use. 0.5 ml each of CLEN and free curcumin dissolved in methanol was poured into the dialysis bag which was then tied at both the ends and placed in a beaker containing 100 ml of dissolution medium maintained at 37 ± 0.5°C and stirred at a rate of 100 rpm. Aliquots of the dissolution medium were withdrawn at different times and replaced with the same volume of fresh medium to maintain the sink conditions. The samples were analyzed spectrophotometrically at 425 nm.

Since curcumin is not stable at pH 1.2 (SGF) and 6.8 (SIF), SGF or SIF were not used for the release study.

### Stability Studies

#### Long Term Stability

CLEN were placed at 5 ± 3°C for evaluating long term stability. Samples were withdrawn at 0, 1, and 3 months interval and evaluated for total drug content, entrapment efficiency, particle size, zeta potential, and PDI.

#### Hydrolytic Degradation Studies

The hydrolytic stability of CLEN and free curcumin was investigated at pH 1.2, phosphate buffer pH 6.8, phosphate buffer pH 7.4, and alkaline borate buffer pH 9 (Indian Pharmacopoeia and Commission, 2014). The stock solution of free curcumin (100 μg/ml) was prepared in methanol by dissolving 5 mg of curcumin in 50 ml of methanol. It was further diluted to 5 μg/ml with respective buffers (pH 1.2, pH 6.8, pH 7.4, and pH 9). In the case of CLEN, 1 ml dispersion was placed in a dialysis bag and dialyzed against methanol (100 ml) at room temperature for 45 min to remove the unentrapped drug. The total drug content (drug assay) of the dialyzed CLEN was determined and diluted suitably with water to prepare a 100 μg/ml stock, which was diluted further to 5 μg/ml with respective buffers. The stock solution and sample solution were prepared in amber colored volumetric flasks to avoid photodegradation. The solutions were prepared and incubated at 37°C. The samples were withdrawn at varying times, viz. 0, 0.5, 1, 2, 4, 6, 8, 10, 12, and 24 h. The samples were then analyzed using the UV/Visible spectrophotometer at λmax 425 nm. The absorbance read at zero time was considered as 100% and change in concentration and percentage degradation was determined accordingly.

The graph between concentration versus time, log concentration versus time, and percent drug remaining versus time were plotted. The degradation constant (k) for the first order was calculated by multiplying the slope of log concentration versus time plot with 2.303.

#### Particle Size Variation With pH and Time

CLEN were diluted (20 times) with buffers of pH 1.2, 6.8, and 7.4 and were incubated at 37°C. Samples were withdrawn at different time intervals and their particle size was determined to establish the stability of CLEN on oral administration.

#### Photostability Studies

The photostability studies on free curcumin (curcumin dispersion in 1% CMC) and CLEN were conducted according to ICH guidelines. Free curcumin and CLEN were stored in both clear glass and amber colored containers. The samples were placed in the photostability chamber and exposed to light providing illumination of not less than 1.2 million lux hours for 10 days. After 10 days, free curcumin was evaluated for total drug content (assay) and CLEN were evaluated for total drug content, entrapment efficiency, particle size, zeta potential, and PDI.

## Bioanalytical Method Validation

Method development and validation of analysis of curcumin in plasma were carried out following U.S. Food and Drug Administration guidelines. The method was validated for system suitability, specificity, sensitivity, recovery, precision, accuracy, and linearity.

A five point calibration curve of curcumin was prepared by spiking 40 μl of blank plasma with 10 μl each of the appropriate working dilution of curcumin to result in 25–500 ng/ml of curcumin.

High quality control (HQC: 500 ng/ml), medium quality control (MQC: 200 ng/ml), and low quality control (LQC: 25 ng/ml) samples were prepared similarly for validation.

### Chromatographic Conditions

The determination of curcumin was carried out using a UPLC system (waters, Acquity UPLC H class). A reversed phase Accucore C18 column (100 mm × 4.6 mm, 2.6 μm; Thermo Scientific, Mumbai) was used. Acetonitrile: Water (1:1, isocratic) was run as the mobile phase and pH was adjusted to 3 with 0.1% acetic acid. The elution was performed at a flow rate of 0.10 mL/min and the analytical column was kept in a thermostatic oven at 35°C. The detection of curcumin was performed with a Waters Photodiode Array Detector at a set wavelength of 425 nm. The injection volume was 10 μL for all standards and samples. Curcumin was eluted approximately 15 min after injection. A series of standard solutions of curcumin (25–500 ng/mL) were prepared in acetonitrile: water.

## Pharmacokinetic Studies

### Study Design

For *in vivo* pharmacokinetic studies, post weaned (4-weeks old) female Wistar Rats weighing 250 g were fasted for 12 h prior to the study. The animals were divided into three groups (*n* = 3). Groups 1 and 2 were administered 100 mg/kg BW of CLEN and CurcuWIN^®^ (a marketed product of curcumin claimed to show enhanced bioavailability), respectively, whereas group 3 was administered 100 mg/kg BW of free curcumin dispersed in 0.5% w/v carboxymethyl cellulose. There are variable reports on the suitable dose of curcumin in animals and several investigators have reported the use of 100 mg/kg dose of curcumin in bioavailability studies and for therapeutic effects (Takahashi et al., 2009; Ravichandran, 2013; Vatsavai and Kilari, 2016). Hence 100 mg/kg dose was used. The blood samples (0.5 ml) were withdrawn from retro-orbital plexus at different time intervals and collected into microcentrifuge tubes containing EDTA. Plasma was separated by centrifuging the blood samples at 10000 rpm for 6 min at 5°C. After centrifugation, the obtained plasma was stored at −20°C until analysis. All animal protocols were approved by the institutional animal ethics committee vide letter number 107/IAEC/18 and approval number PU/45/99/CPCSEA/IAEC/2017/89.

### Sample Preparation (Extraction Procedure)

To 40 μl of plasma samples in an eppendorf tube, 150 μl of methanol and 300 μl of acetonitrile:water (1:1; pH 3) was added. 10 μl of the β-glucuronidase enzyme was added and incubated for 30 min at 37°C. The sample was vortexed for 5 min and centrifuged at 15,000 rpm to separate precipitated proteins. The supernatant was transferred to suitably labeled tubes.

The sample was filtered through a 0.2 μm syringe filter and was used for analysis using the developed UPLC method. All the conditions of UPLC were maintained as indicated under section “Chromatographic Conditions.”

### Data Analysis

The pharmacokinetic parameters were calculated using a non-compartmental model. The area under the concentration-time curve from time zero to time t (AUC_0–t_) was calculated using the trapezoidal method. Peak concentration (C_max_) and the time at which the peak concentration is achieved (T_max_), were obtained directly from the individual concentration-time profiles. The area under the concentration-time curve from time zero to infinity was calculated by AUC_0__–__∞_ = AUC_0__–__t_ + C_t_/K_e_, where C_t_ is the drug concentration observed at the last time and K_e_ is the apparent elimination rate constant obtained from the terminal slope of the individual concentration-time curves after logarithmic transformation of the concentration values and application of linear regression. AUMC was determined by plotting concentration × time (ct) versus time (t) using the trapezoidal method. Mean residence time (MRT) was calculated as = AUMC/AUC.

## Results and Discussion

### Curcumin Loaded Solid Lipid Nanoparticles (CLEN)

Various formulations incorporating 10–15 mg (1–1.5%) of curcumin per ml of CLEN dispersion were prepared, as described in Supplementary Table S1. Most of these SLN systems, however, showed settling of curcumin crystals at the bottom of the SLN CLEN formulation within 24 h of preparation, upon keeping.

All the batches prepared with Compritol^®^ 888 ATO with concentration varying from 4 to 10% w/w showed settling of the drug. However, no settling was observed when Precirol ATO 5^®^ was used as the lipid component (F4 and F5). Howsoever, F5 formulation showed a particle size of ≥1 μ (1000 nm), hence, three batches of F4 were prepared and evaluated for TDC, entrapment efficiency, and particle size (Supplementary Table S2).

The particle size of the selected formulation F4 was also >700 nm. In the next part of the study, an attempt was made to decrease the particle size of the formulations F4 and F5 by varying stirring speeds and HPH cycles as shown in Supplementary Table S3.

It was observed that an increase in stirring speed and number of HPH cycles was not successful in achieving a particle size of <700 nm. Furthermore, F4 and F5 also showed settling of curcumin upon longer keeping (>2 weeks) and were thus rejected.

As the next option, we decided to combine Compritol 888 ATO^®^ with GMS (lipid mixture) in different ratios to prepare CLEN formulation and the prepared formulations were observed for the settling of curcumin (Supplementary Table S4). It has been reported that curcumin showed maximum solubility in GMS when a panel of lipids was evaluated (Shrotriya et al., 2018). Our earlier studies indicated that lipid mixtures show greater imperfections and reduced crystallinity, allowing for better encapsulation of drug molecules (Bhandari and Kaur, 2013). Compritol 888 ATO^®^ is a safe pharmaceutical excipient which results in nanosized and stable SLN formulations, in addition to the fact that curcumin shows some solubility in it (Shrotriya et al., 2018). Hence, we decided to combine GMS with Compritol 888 ATO^®^ in the next set of curcumin SLN formulations (Supplementary Table S4).

Since no settling of the drug was observed, the prepared formulations (F8-F13) were evaluated for drug assay/TDC, entrapment efficiency, particle size, and PDI, as described in Supplementary Table S5.

Formulations (F8-F10) showed the presence of curcumin crystals when observed under a light microscope, indicating that the drug is present in undissolved/undispersed form, and was hence rejected. Out of the remaining three formulations (F11, F12, and F13), F13 was selected based on the fact that it achieved the highest drug concentration (15 mg/ml).

In the present investigation, we developed CLEN (curcumin encapsulated lipidic nanoconstructs; SLNs) dispersion (Figure 1), with a high drug loading (15% with respect to lipid matrix), and containing 15 mg curcumin per ml. It is the highest increase in solubility of curcumin in an aqueous system (1.4 × 10^6^ times increase as compared to 11 ng/ml in water for free curcumin) coupled with high drug loading, reported till date, to the best of our knowledge.

Supplementary Table S6A summarizes features of curcumin SLNs reported previously in the literature and the technical advantage of the presently disclosed CLEN technology (Indian patent application no. 201811044487; PCT/IB2019/060162) and Supplementary Table S6B summarizes curcumin loaded nanoparticles (other than SLNs) investigated over a period of 5 years.

### Characterization of CLEN

The TDC/assay of CLEN was found to be 14.62 ± 0.14 mg/ml (97.46 ± 0.01%) (*n* = 6). The entrapment efficiency was 79.74 ± 0.88% (*n* = 6), and the average particle size was 540.2 ± 45.1 nm (*n* = 6). The average particle size of blank SLNs was 339.6 nm. A much larger particle size upon loading of curcumin indicates that curcumin is probably surface loaded in addition to being incorporated in the core of SLNs. The latter could be due to the high solubility of curcumin in the surfactant layer surrounding the SLNs. Tween 80 is presently used as the surfactant and PEG, though used as a solvent, is also known for its surfactant supporting properties and curcumin shows high solubility in both these components.

The PDI of CLEN and blank SLNs was 0.291 ± 0.062 (*n* = 6), and 0.332, respectively. While PDI of ≤0.3 is considered to be highly monodisperse, values of 0.3–0.4 are considered to be moderately polydisperse (Bhattacharjee, 2016).

The zeta potential of CLEN and blank SLNs was −10.88 ± 3.67 mV (*n* = 6) and −0.61 mV, respectively. When curcumin was loaded, the potential value of the particles decreased, which may be due to the free curcumin distributed in the water phase or potential diffusion layer (Xu et al., 2016) and can be attributed to the phenolic (OH^–^) and diketonic functionalities of the curcumin (Pathak et al., 2015).

#### Field Emission Scanning Microscopy (FESEM)

**FIGURE 2:**
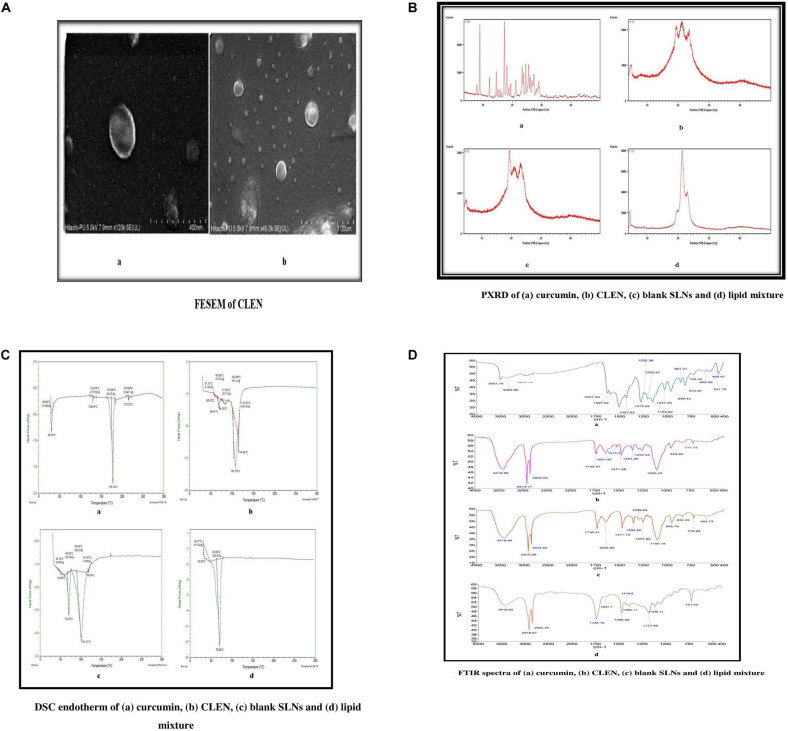
**(A)** FESEM, **(B)** PXRD, **(C)** DSC, and **(D)** FTIR of CLEN and other components.

The FESEM of CLEN showed that the particles were nearly spherical in shape and that they were present as individual entities rather than agglomerates, confirming their stability. An outer coating of the surfactant is observed in Figure 2A. The surfactant layer assigns stability to the particles preventing their aggregation.

#### Powder X-Ray Diffraction

Powder X-ray diffraction patterns of curcumin, CLEN, blank SLNs, and lipid mixture are shown in Figure 2B. The PXRD pattern of curcumin exhibited peaks at 2θ scattered angles of 17.27°, 8.89°, 25.57°, and 24.54°, indicating its crystalline nature. The PXRD pattern of lipid mixture exhibited sharp peaks at 2θ scattered angles 21.31°, 22.96°, 4.46°, and 19.63°, again, establishing its crystalline state (Figure 2B). CLEN and blank SLNs, however, showed spectra differences from curcumin and the lipid mixture. Some characteristic peaks observed at 2θ scattered angles of 21.2°, 19.3°, and 23.5° in case of CLEN and 19.33°, 21.12°, and 22.95° in case of blank SLNs do correspond to the lipid mix. However, the typical pattern of peaks corresponding to free curcumin was missing, indicating loss of crystallinity and a shift toward the amorphous (Wang et al., 2012) and thus a more soluble state.

#### Differential Scanning Calorimetry (DSC)

Differential scanning calorimetry is a thermoanalytical technique in which the differences in the amount of heat required to maintain the sample and reference at the same temperature is measured as a function of temperature and time. In the case of pure curcumin, a melting endotherm appeared at 178.19°C corresponding to its reported melting point at 180–183°C. The CLEN, however, showed a broad endotherm starting from 60°C and extending to 115°C, with a sharp peak at 107.79°C (Figure 2C). The broadening of peaks indicates the amorphous nature of CLEN while the shift to a lower temperature indicates a nano size. The lipid mixture exhibited a sharp peak at 70.68°C, which was, however, absent in CLEN. This may be due to the change of lipid to a completely amorphous form in SLN formulation (Chen et al., 2013).

#### Fourier Transform Infra-Red (FTIR)

Pure curcumin samples showed a sharp peak at 3507 cm^–1^ indicating the presence of –OH group (Figure 2D). The IR peaks obtained with CLEN, however, revealed an intermolecular stretching of the –OH groups (3400–3200 cm^–1^) due to hydrogen bonding (Shelat et al., 2015). This may be regarded as a direct indication of the formation of SLNs, as the 3400–3200 cm^–1^ stretching was not observed in the case of curcumin or lipid mixture.

### *In vitro* Release

The selection of a suitable release media was of significance with curcumin considering its insoluble nature and proneness to degradation in various buffer solutions. Curcumin shows significant solubility in methanol, so varying proportions of methanol were mixed with solutions buffered to pH 1.2 (stomach), 6.8 (proximal intestine), and 7.4 (distal part of small intestine/plasma). However, the addition of methanol led to precipitation of electrolytes from buffers. The mixing of ethanol with the buffer solutions overcame this problem, but curcumin was found to degrade in these buffer solutions. Hence, methanol:water (1:1) was selected as a suitable release medium. Varying concentrations of methanol (10–50%) were tried, however, only 50% methanol in water could dissolve a sufficient quantity of curcumin (present in 0.5 ml of CLEN dispersion), to ensure sink conditions.

**FIGURE 3:**
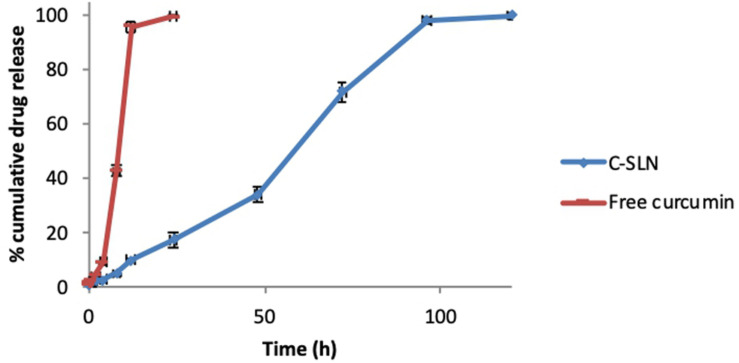
*In vitro* release profile of curcumin from CLEN and free curcumin.

The drug release from CLEN and free curcumin is shown above in Figure 3. The release from CLEN extended up to 120 h with 99.73 ± 1.12% release (Figure 3) and followed zero order release kinetics, i.e., controlled release (Table 1). A first order release was observed as expected with free curcumin and the drug was completely released within 24 h (Table 1).

**TABLE 1:** Linear correlation coefficients obtained for *in vitro* release data from various models.

Model		Formulation	
		Free curcumin	CLEN
**Zero order**	***r*^2^**	0.856	0.978
**First order**	***r*^2^**	0.964	0.855
**Higuchi**	***r*^2^**	0.879	0.917
**Hixson-Crowell**	***r*^2^**	0.906	0.933
**Korsmeyer-peppas**	***r*^2^**	0.957	0.969
	**n**	1.149	0.841

### Stability Studies

#### Long Term Stability

**FIGURE 4:**
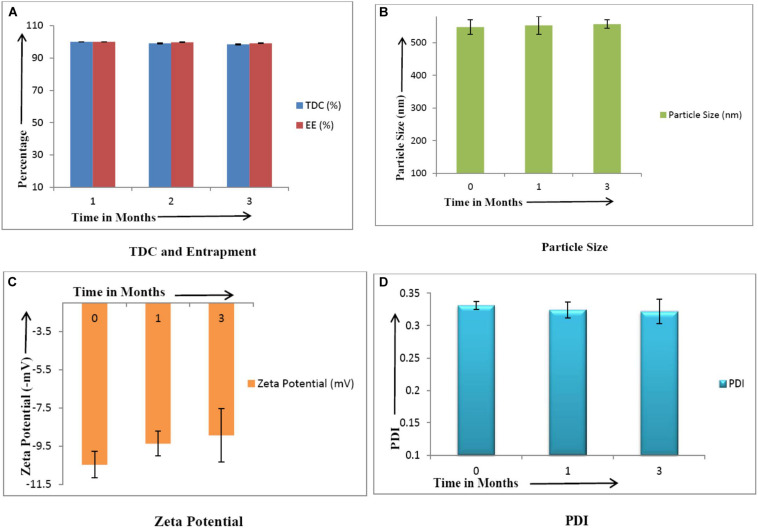
Stability studies of CLEN (*n* = 3). There was no significant change (*p* ≤ 0.05) in the values of various parameters upon storage. **(A)** TDC and Entrapment, **(B)** Particle Size, **(C)**, Zeta Potential, and **(D)** PDI.

After 3 months of storage under refrigerated conditions, CLEN was found to be stable with no significant change (*p* ≤ 0.05) in any of the parameters (Figure 4).

#### Hydrolytic Degradation

**FIGURE 5:**
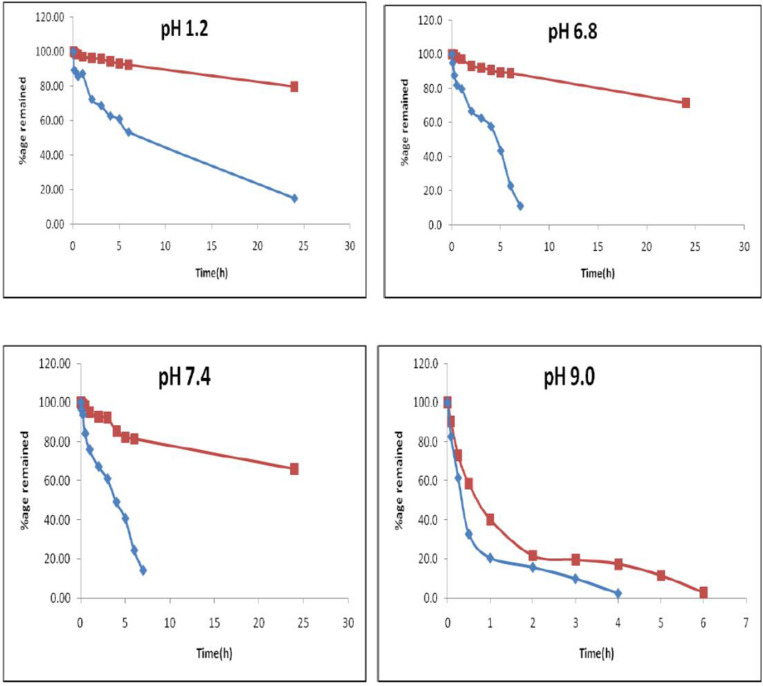
Percentage remaining of curcumin versus time plot at various pH.

The study aimed to investigate if the encapsulation of curcumin within CLEN protects against hydrolytic degradation. Data from free curcumin was compared with those for CLEN (Table 2 and Figure 5).

**TABLE 2:** Percentage remaining of free curcumin (F.Cur) and CLEN with time at various pH (*n* = 3).

Time	pH 1.2		pH 6.8		pH 7.4		pH 9	
(h)	F.Cur	CLEN	F.Cur	CLEN	F.Cur	CLEN	F.Cur	CLEN
0	100.00 ± 0.06	100.00 ± 0.03	100.00 ± 0.04	100.00 ± 0.06	100.0 ± 0.060	100.00 ± 0.06	100.00 ± 0.004	100.00 ± 0.03
0.08	89.23 ± 0.02	99.49 ± 0.04	94.93 ± 0.06	99.80 ± 0.01	96.33 ± 0.02	99.49 ± 0.05	82.59 ± 0.009	90.45 ± 0.004
0.25	88.29 ± 0.01	98.87 ± 0.03	87.63 ± 0.04	99.49 ± 0.02	93.81 ± 0.04	98.87 ± 0.04	61.43 ± 0.004	72.87 ± 0.006
0.5	85.60 ± 0.01	98.25 ± 0.03	81.95 ± 0.08	98.08 ± 0.02	84.17 ± 0.04	98.25 ± 0.07	32.76 ± 0.006	58.39 ± 0.006
1	87.24 ± 0.001	97.12 ± 0.03	79.72 ± 0.06	97.06 ± 0.01	75.92 ± 0.04	95.07 ± 0.07	20.48 ± 0.001	40.13 ± 0.003
2	72.37 ± 0.02	96.20 ± 0.03	66.73 ± 0.05	93.22 ± 0.03	67.20 ± 0.03	92.60 ± 0.06	15.70 ± 0.002	21.66 ± 0.005
3	68.74 ± 0.03	95.79 ± 0.03	62.68 ± 0.07	92.21 ± 0.03	61.01 ± 0.03	92.29 ± 0.05	9.90 ± 0.003	19.53 ± 0.005
4	62.76 ± 0.01	94.35 ± 0.03	57.81 ± 0.05	90.79 ± 0.02	49.08 ± 0.04	85.51 ± 0.07	2.39 ± 0.004	17.41 ± 0.006
5	61.01 ± 0.01	93.11 ± 0.03	43.61 ± 0.08	89.37 ± 0.03	40.60 ± 0.03	82.32 ± 0.06	–	11.46 ± 0.003
6	53.40 ± 0.02	92.39 ± 0.04	23.12 ± 0.04	89.07 ± 0.02	24.31 ± 0.01	81.50 ± 0.05	–	2.76 ± 0.006
24	15.76 ± 0.01	79.86 ± 0.08	11.36 ± 0.04	71.56 ± 0.02	13.99 ± 0.01	66.08 ± 0.05	–	–

From the data generated in this study, it is observed that at acidic pH 1.2, CLEN did not show any significant degradation (approximately 8%) up to 6 h (*p* < 0.05). However, nearly 20% of curcumin entrapped within the SLNs degraded at 24 h (*P* < 0.001). In contrast, degradation of up to 50% and 85% is observed in 6 and 24 h, respectively for free curcumin. At pH 6.8, 77% free curcumin degraded within 6 h and 89% degraded within 24 h, whereas in the case of the entrapped drug (CLEN), we observed only a 10% degradation up to 6 h and 30% degradation up to 24 h. Similarly, at physiological pH 7.4, a significant amount (75%) of free curcumin was degraded within 6 h and 77% degraded in 24 h and at extreme pH 9, free curcumin showed approximately 98% degradation at 4 h while the same amount of curcumin degraded at 6 h. The data demonstrate that SLNs (red line for CLEN and blue line for curcumin) significantly protect the encapsulated curcumin against hydrolytic degradation (Figure 5).

The degradation kinetics of curcumin under various pH conditions and the stability of curcumin in physiological matrices reported earlier indicates that when curcumin was added to a 0.1 M phosphate buffer (pH 7.2), more than 90% of curcumin was degraded (Wang et al., 1997). The absorbance at 426 nm decreased to approximately 50% after 5 min, and after 10 min the remaining absorbance was only about 10%. However, we presently observed an almost 40% degradation at 4 h in pH 1.2 medium.

The order of kinetics of free curcumin was zero order at all investigated pH conditions, while in the case of CLEN, the order was first order except at pH 9 (zero order). However, even at pH 9, t_1/2_ increased five times, and the rate constant of CLEN was 80% less than that for free curcumin. Very interestingly, at a physiological pH of 7.4, an almost 90% decrease in *k* value and a 10 times increase in t_1/2_ was observed (Table 3). Similarly, protection provided at the other two pH buffers was also substantial and a similar increase in t_1/2_ (13.3 times at pH 1.2 and 104 times at pH 6.8), and a decrease in k was observed in comparison to free curcumin.

**TABLE 3:** Various degradation kinetics parameters for free curcumin and CLEN (*n* = 6).

Order	pH	Free cur			C-SLNs		
		
		*k* value	*^t^*1/2	*r*^2^	*k* value	*^t^*1/2	*r*^2^
**zero order**	pH 1.2	0.039	17.34	0.934	–	–	–
	pH 6.8	0.52	1.32	0.967	–	–	–
	pH 7.4	0.073	9.4	0.984	–	–	–
	pH 9	0.95	0.669	0.994	0.195	3.55	0.891
**First order**	pH 1.2	–	–	–	0.003	231	0.98
	pH 6.8	–	–	–	0.005	138	0.962
	pH 7.4	–	–	–	0.007	99	0.89

#### Particle Size Variation With pH and Time

Variation in particle size at the above mentioned physiological pH was determined at times corresponding to their residence times in these parts of the gastrointestinal tract (g.i.t.). No significant (*p* ≤ 0.05) change in size and PDI (Table 4) was observed at pH 1.2 and 6.8 indicating the stability of CLEN upon incubation at these pH values. This ensures that CLEN will be absorbed in the nano form from the g.i.t. Furthermore, at pH 7.4, even after incubation for 24 h, only a 6.7% increase in size was observed. It may thus be concluded that CLEN remain stable and maintain their integrity under these physiological conditions.

**TABLE 4:** Change in particle size and PDI of CLEN with pH.

Dilution medium	Time (h)	Particle size# (nm)	PDI#
**pH 1.2**	0	458.9 ± 8.8	0.361 ± 0.012
	2	447.9 ± 7.4	0.352 ± 0.030
	4	489.3 ± 7.3	0.363 ± 0.013
**pH 6.8**	0	453.0 ± 20.5	0.320 ± 0.011
	2	434.3 ± 3.7	0.369 ± 0.012
	4	453.0 ± 17.0	0.363 ± 0.003
	6	475.1 ± 8.7	0.398 ± 0.006
**pH 7.4**	0	465.9 ± 12.5	0.332 ± 0.012
	24	596.7 ± 39.0*	0.341 ± 0.017

#Particle size and PDI for the CLEN aqueous dispersion used in the experiment were 450.1 nm and 0.331, respectively. No significant change in particle size was observed at any pH or time except for those marked with * (p ≤ 0.05).

#### Photostability Studies

**FIGURE 6:**
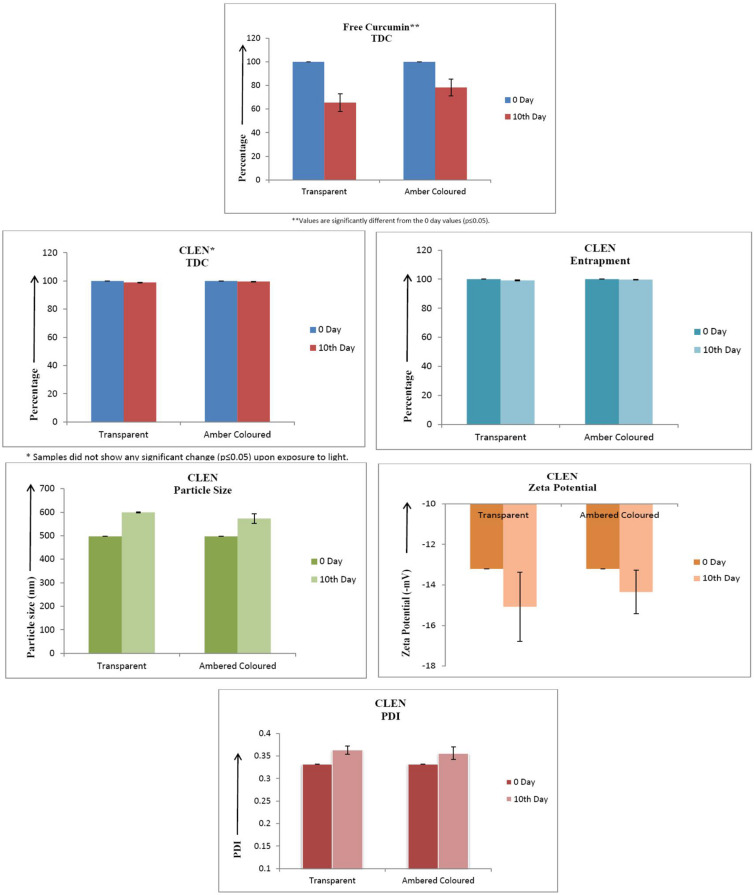
Photostability studies on various parameters of CLEN and its comparison with free curcumin (*n* = 3).

The study was undertaken to establish the photoprotection offered to curcumin by the lipid matrix of CLEN. It protected the encapsulated curcumin completely against photodegradation. On the other hand, 21.7% of degradation was observed upon storage of free curcumin in amber colored containers while it increased to 35% in transparent containers (Figure 6). No degradation (TDC/assay remained unchanged) occurred in the case of CLEN even upon storage in transparent containers. However, an increase in particle size was observed upon exposure to light. This may be attributed to increased collisions between particles due to imparted kinetic energy by light.

### Bioanalytical Method Validation

A calibration curve of curcumin was found to be linear from 12 to 500 ng/ml in plasma samples. The system was confirmed to be suitable, selective, and specific for the determination of curcumin under the optimized chromatographic conditions, as no peak was observed in the chromatograms of blank plasma samples.

Limit of detection (LOD) and LLOQ were found to be 2 ng/ml and 25 ng/ml, respectively. The overall recovery was >80%. The individual recoveries at LQC, MQC, and HQC were 82.18, 86.18, and 83.42%, respectively.

The intra-day accuracy was 98.43–112.21% for QC samples with precision <2% and the inter-day accuracy was 99.17–107.83% for QC samples with precision <2.6%.

### Pharmacokinetic Studies

**FIGURE 7:**
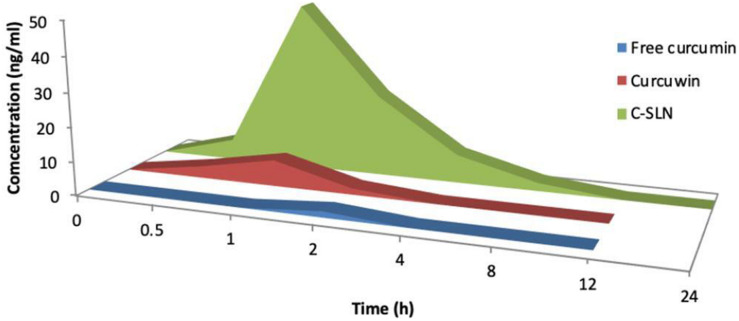
Mean concentration-time area curve of curcumin rat after single oral does of free curcumin, curcumin^®^ and CLEN.

Plasma concentration after oral administration of 100 mg/kg dose of CLEN and CurcuWIN^®^ were compared with 100 mg/kg dose of free curcumin and plotted against time (Figure 7). The area under the curve was calculated using the trapezoidal method. The relevant parameters including C_max_, T_max_, AUC_0__–__∞_, and clearance are listed in Table 5 above.

**TABLE 5:** Various pharmacokinetic parameters obtained after a single oral dose of free curcumin, CurcuWIN^®^ and CLEN administered to rats (*n* = 3).

Formulation	Dose (mg/kg)	^AUC^0–∞ (h×ng/ml)	Cmax (ng/ml)	Tmax (h)	^AUMC^0–∞ (h^2^×ng/ml)	MRT (h)	Clearance (l/h/kg)	Relative bioavailability with respect to Free curcumin
**Free curcumin**	100	1.78	1.18	2	3.55	2	56287.29	1
** CLEN**	100	124.2	55.75	1	401.28	3.20	857.62	69.78
**CurcuWIN^®^**	100	16.02	8.52	1	20.43	1.27	6240.56	9.00

The studies revealed that relative bioavailability of free curcumin was increased by 69.78 times in the case of CLEN, whereas it was increased by only 9.00 times in CurcuWIN^®^. In a pharmacokinetic study performed on human volunteers, CurcuWIN^®^ showed 136 times higher bioavailability than free curcumin (Jäger et al., 2014). From this, we can imply that CLEN may also show a bioavailability of 1047 times (136 × 7.7) when determined in humans.

**FIGURE 8:**
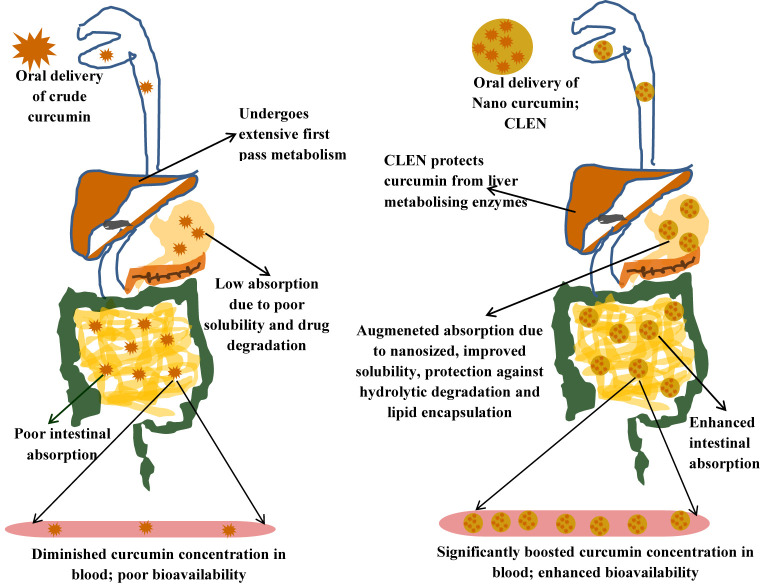
Diagrammatic representation for benefits of encapsulation of curcumin as CLEN.

Figure 8 shows the advantage of encapsulating curcumin as CLEN and its benefits at various sites in the physiological system when taken orally. Wei et al. (2014) formed a nanogel of curcumin using cholesteryl-hyaluronic acid which showed an improved circulation, slower clearance as observed by authors in a pharmacokinetic study in mice. We also observed a significantly slower clearance in the CLEN group (Table 5). The nanogel formulation showed excellent tumor growth inhibition in human pancreatic MiaPaCa-2 xenograft (5 fold) and murine mammary 4T1 orthotopic cancer models (2.5 fold) when analyzed for mean tumor volume with respect to the free curcumin group (Wei et al., 2014).

## Conclusion

This study was successful in formulating CLEN containing 15 mg curcumin per ml of the SLN dispersion. The characterization of the prepared formulation helped us to conclude that the SLNs were formed successfully and were amorphous, confirming the presence of curcumin in a solubilized form.

Furthermore, CLEN exhibited a-zero order release in comparison to first order release by free curcumin, indicating the controlled release nature of the developed CLEN. Stability studies including photostability and hydrolytic degradation studies confirm the stability of CLEN and curcumin encapsulated within the lipid matrix.

Previous reports by numerous scientists have proved the regulatory effect of curcumin on NF-kB and its cytotoxic effect on cancer cell lines. The only limiting factor was the availability of curcumin in the physiological system to elicit a therapeutic response. This is suitably addressed by the presented CLEN formulation. With enhanced solubility, stability, permeability, and bioavailability, curcumin prepared CLEN could be explored for various therapies including cancers and other inflammation and oxidative stress related pathologies in the future.

## Data Availability Statement

The raw data supporting the conclusions of this article will be made available by the authors, without undue reservation.

## Ethics Statement

The animal study was reviewed and approved by the Institutional Animal Ethical Committee Panjab University, Chandigarh.

## Author Contributions

IPK conceived the idea and developed the theory. TG, JS, and SK carried out the experiments. SS helped in the synthesis of CLEN and performed the analytic calculations under the supervision of IPK and GS. JS wrote the manuscript with support from SS. All authors contributed to the article and approved the submitted version.

## Conflict of Interest

The authors declare that the research was conducted in the absence of any commercial or financial relationships that could be construed as a potential conflict of interest.
