# Supplementary material for: Enhancing Bioavailability and Stability of Curcumin Using Solid Lipid Nanoparticles (CLEN): A Covenant for Its Effectiveness
Source: Front Bioeng Biotechnol. 2020 Oct 15;8:879. doi: 10.3389/fbioe.2020.00879 (PMC7593682; doi:10.3389/fbioe.2020.00879)
Supplement: Supplementary file 1 [file Data_Sheet_1.pdf]

Supplementary data sheet

Table 1: Composition of curcumin loaded solid lipid nanoparticles (CLEN) with high curcumin concentration (1-1.5%)

Formulation code	Lipid (%)	Tween 80 (%)	PEG 600 (%)	Phospholipon 90 G (%)	Curcumin (%)	Drug settling
F1	4	12	8	0.4	1	✓
F2	6	12	8	0.4	1	✓
F3	6	12	8	0.8	1	✓
F4*	6	12	8	0.4	1	x
F5*	6	12	8	0.4	1.5	x
F6	8	12	8	0.8	1	✓
F7	10	12	8	0.8	1	✓

*All formulations contained Compritol 888 ATO® as the lipid component except F4 and F5 which contained Precirol ATO 5®. ✓ indicates settling and x indicates no settling.

Table 2: Characterisation of F4 formulation

F4	TDC	Entrapment efficiency	Particle size (nm)
1	89.3%	67.52%	> 700
2	89.7%	65.66%	> 700
3	90.3%	67.88%	> 700

Table 3: Variation of stirring speed and HPH cycles

S.NO.	Drug concentration (%w/v)	Stirring speed (rpm)	HPH cycles	Particle size (nm)	PDI
1	1%	8000	3	1000	0.384
2	1%	8000	4	699.8	0.320
3	1%	8000	5	913.0	0.371
4	1%	8000	6	711.3	0.424
5	1%	9000	3	841.5	0.338
6	1%	9000	4	885.0	0.337
7	1%	9000	5	944.5	0.358
8	1%	9000	6	639.7	0.344
9	1%	10000	3	609.8	0.407
10	1%	10000	4	747.5	0.399
11	1%	10000	5	777.9	0.390
12	1%	10000	6	699.0	0.406
13	1.5%	8000	3	1186.6	0.383
14	1.5%	8000	4	1080.1	0.368
15	1.5%	8000	5	1031.3	0.466
16	1.5%	8000	6	901.3	0.321

Table 4: Formulation development of CLEN using lipid mixture

Formulation Code	Compritol 888 ATO® (%)	GMS (%)	Tween 80 (%)	PEG 600 (%)	Phospholipon 90 G (%)	Curcumin (%)	Drug Settling
F8	3	3	12	8	0.4	1.5	x
F9	4	2	12	8	0.4	1.5	x
F10	6	2	12	8	0.4	1.5	x
F11	8	2	12	8	0.4	1.0	x
F12	8	2	12	8	0.4	1.2	x
F13	8	2	12	8	0.4	1.5	x

GMS- glyceryl monostearate; x- no settling

Table 5: Characterization of CLEN formulations

Formulation code	Particle size (nm)	PDI	TDC (mg/ml)	Entrapment efficiency(%)
F8	975.2	0.376	13.87	82.29
F9	556.5	0.315	13.78	84.83
F10	598.2	0.373	13.83	87.76
F11	473.5	0.087	09.29	82.13
F12	564.1	0.354	11.4	85.60
F13	538.8	0.369	14.35	82.90

Table 6: List of previous scientific reports of curcumin solid lipid nanoparticles and the technical advantage of CLEN over these inventions

S No.	SLNs reported earlier	Organic solvent	Composition	DL, EE and other characteristics (stability & kinetics data)	Technical advantage of CLEN	Reference
**1.**	Nanoemulsion technique employing high-speed homogenizer and ultrasonic probe	Not used	Aqueous phase- Polaxamer, Tween 80.<br>Lipid phase - Soya lecithin PC, Trimyristin or Tristearin or Glyceryl monostearate.	DL: 0.2% w/w<br>DC: 0.3% w/w<br>PS: 109-203 nm<br>PDI: 0.167-0.210.<br><br>No pharmacokinetic studies done.<br>Stability over 6 months at room temperature.	DL as well as DC is much higher	(Nayak *et al.*, 2010)
**2.**	Hot melt oil-in-water (o/w) emulsion technique	Dicholorom ethane	Lipid phase - Stearic acid.<br>Aqueous phase- Poloxamer.	PS: 250 nm<br>EE: 69%.<br><br>No kinetics reported.<br>Exhibited stability	Organic solvent is used in the cited formulation.<br>EE is higher.	(Sutaria *et al.*, 2012)

				at lower temp (4ºC & 24ºC) and agglomeration at 37ºC.		
**3.**	High speed homogenisation followed by ultrasonication and low temperature solidification	Not used	Aquoeus phase- Lutrol F68, Tween 80. Lipid phase- Precirol ATO 5 Miglyol 812.	DL= 5.88% w/w PS= 162.4 ± 10.5 nm EE= 87% No kinetics and stability data	DL is higher. High pressure homogenisation is a more industry amenable process.	(Puglia *et al.*, 2012)
**4.**	Emulsification followed by high speed homogenization	Not used	Aqueous phase- Poloxamer. Lipid phase- Monolein Sodium cholate.	DL: 0.33% w/w DC: 0.015% w/w PS: 190nm No stability data C_{max}: 20.85μM (IP dose @ 400mg/kg) AUC 26 fold increase compared to free curcumin	DL as well as DC is much higher Superior kinetics of CLEN	(Wang *et al.*, 2012)
**5.**	Nanoemulsion technique employing high-speed homogenizer and ultrasonic probe	Not used	Aquous phase- Tween 80. Lipid phase- Glyceryl monostearate, Oleic acid, lecithin.	DL: 1.5 % w/w DC: 0.006 w/v PS: 108 nm PDI: 0.28 EE: 78% No kinetics data Stability in simulated gastric medium at 92% after 6 h.	DC is much higher	(Aditya *et al.*, 2013)
**6.**	Hot high pressure homogenisation	Ethanol	Aquoeus phase- Pluronic F-68. Lipid phase- Dynasan 114® and Sefsol-218.	DL: 0.74% w/w DC: 8mg/100ml PS: 145 nm PDI: 0.213 EE: 92.34% Relative bioavailability of 125% in comparison to free curcumin given at 2 mg/kg bolus i.v.	DL as well as DC is much higher	(Sun *et al.*, 2013)
**7.**	High speed homogenization followed by low temperature solidification	Not used	Aqueous phase- Taurocholate. Lipid phase- Stearic acid Lecithin.	DL: 1.1 %w/w PS: 148 nm No stability and kinetic data	DL is higher	(Sandhir *et al.*, 2014)

**8.**	High pressure homogenization followed by ultracentrifugation	Not used	Aqueous phase- Propylene glycol. Lipid Phase- Compritol 888ATO or PrecirolATO 5, Lipoid S 75.	DL: 5% w/w DC: 1% w/w PS: 200-300 nm Formulation stored at 5±3 °C for 1 year found to be stable Encapsulation of Curcumin into SLNs led to 12 fold increase in bioavailability at dose of 50mg/kg given orally.	DC is higher Increase in bioavailability is more	(Shelat *et al.*, 2015)
**9.**	Emulsification and low temperature solidification method	Chloroform	Lipid phase- Stearic acid, lecithin. Aqueous phase- Myrj52.	DL: 36% w/w PS: 40-80nm No stability and kinetic data	Chloroform has been used in cited formulation whereas no organic solvent has been used in our formulation	(Wang *et al.*, 2015)
**10.**	High pressure homogenization method	Not used	Aqueous phase- Tween 80 and Kolliphor® P188. Lipid Phase- Precirol ATO®5, Miglyol 812N/F.	DL: 3% w/w DC: 0.3% w/w PS: 280nm PDI: 0.4 No stability and kinetic data	DC is much higher	(Beloqui *et al.*, 2016)
**11.**	Modified emulsion/solvent evaporation	Ethanol	Aqueous phase- Tween 80. Lipid Phase- Glyceryl monostearate/ stearic acid/ ceramide.	DL: 14%w/w PS: 102-156 nm PDI: 0.187-0.428 Ceramide SLNs (C-SLNG-3) showed maximum stability for 180 days C_{max} was also highest with C-SLNG-3 and absolute bioavailability of 68.12%.	Organic solvent is used in the cited formulation. DL is higher. Oral bioavailability enhancement of 69.78 times (6978%)	(Gaur *et al.*, 2016)
**12.**	Hot high-pressure homogenization	Not used	Aqueous phase- Tween 80 and soya lecithin. Lipid phase- Precirol	PS: 146nm PDI: 0.189 EE: 90.86% ZP: -21.4 Relative	Lower concentration of lipid and surfactants is used in	(Madane and Mahajan, 2016)

			ATO®5 and capmul MCM.	Bioavailability of more than 400% in comparison to curcumin suspension after intranasal administration<br>No stability data	CLEN.<br>Bioavailability enhancement is 6978%	
**13.**	Emulsification and low temperature solidification method	Ethyl acetate and ethanol	Aqueous phase- Brij78 and TPGS.<br>Lipid phase- Glyceryl monostearate and soya lecithin.	DL: 15% w/w<br>DC: 0.1 % w/v<br>PS:135.3 nm<br>Kinetic: Intragastric at dose of 50 mg/kg, C_{max} with Curcumin SLNs found to be 3.1 folds higher and extended T_{max} in comparison to curcumin<br>No stability data	DC is higher.<br>Organic solvents not used in our formulation.<br>C_{max} was 47.2 times higher	(Ji *et al.*, 2016)
**14.**	Solvent injection method	Chloroform	Aqueous phase- Myrj 52.<br>Lipid phase- Stearic acid and lecithin.	DL: 28%<br>PS: 190.4 nm<br>PDI: 0.286<br>EE: 75%<br>Stability study at 4°C for 1 month with no deviation.<br>No kinetic data	Chloroform is used	(Righeschi *et al.*, 2016)
**15.**	High pressure homogenization	Ethanol & acetone	Lipid phase: cholesterol<br>Aqueous phase: Tween 80	PS: 112-163nm<br>EE: 71%<br>No stability and kinetic data	Ethanol & acetone is used in cited literature	(Jourghanian *et al.*, 2016)
**16.**	Emulsification and low temperature solidification method	Chloroform	Lipid phase: stearic acid, lecithin<br>Aqueous phase: Myrj52	DL: 23.38%<br>EE:72.47%<br>PS: 30-50nm<br>No stability and kinetic data	Chloroform is used	(Wang *et al.*, 2018)
**17.**	High shear homogenization and ultrasonication techniques	Not used	Lipid phase: precirol, palmitic acid and gelucire<br>Aqueous phase: Tween 80	PS: 86.60nm<br>PDI: 0.29<br>EE: 98.9%<br>ZP: -22.15Mv<br>No stability and kinetics data	Complex formulae and a higher concentration of lipid is used in cited literature	(Ganesan *et al.*, 2019)
**18.**	Oil in water emulsion technique using high speed blender followed by sonication	Ethanol	Lipid phase: Tristearin<br>Aqueous phase: polyoxyethylene	DL: 5% w/w<br>EE: 91.15%<br>PS: 242nm<br>ZP: -8.80mV	No organic solvent used	(Bane *et al.*, 2020)

			(10) stearyl ether (PEG10SE), polyoxyethylene (1 0 0) stearyl ether (PEG100SE)	No Stability data Cmax of 108ng/ml and increased AUC with SLN containing PEG100SE @ 50 mg/kg		

DL: drug loading, DC: drug content (assay), PDI: polydispersity index, EE: entrapment efficiency, PS: particle size, ZP: zeta potential

Table 6b: List of curcumin loaded nanoparticles investigated over last 5 years.

S.No	Type of nanoparticles	Characteristics	Targeted disease condition	Competitive edge of CLEN	Reference
1.	Dendrimers (D)	- HA conjugated PAMAM -D<br>-DL:17.26%<br>-EE: 24.54%<br>-PS :9.3 ± 1.5 nm<br>-ZP: -7.02mV	Pancreatic cancer	- EE is higher<br>-DMSO used in cited literature versus no organic solvent used in CLEN	*Kesharwani, P., et al 2015*
2.		- MUC-1 targeted PEGylated Au-D<br>-DL: 7.13%<br>-EE: 82%	Colorectal adenocarcinoma	-DL is higher<br>-methanol used as organic solvent in cited literature	*Alibolandi,M., et al 2018*
3.		-G4 PAMAM -D-Palmitic acid core-shell NP<br>-EE: 80.87%	Acute stress	-Researchers have used commercially available dendrimers, whereas CLEN is in-house prepared simple, industry amenable technology.	*Tripathi, P. K., et al 2020*
4.	Polymeric Micelles (PM)	- GA-Cur PMs<br>-PS:270nm<br>-ZP:-36mV	Hepatocellular & breast carcinoma	-Complex formulae	*Sarika, P.R., et al 2015*
5.		-Cur-TPGS-PMs using	Chronic breast cancer therapy	Complex formulae,	*Ji S, et al 2018*

		methanol<br>-EE: 93.17%<br>-PS: 60.76nm		organic solvent and a high concentration of surfactants used in cited literature	
6.		-Peptide Cur PM in DCM<br>-EE: 76%<br>-PS: 49nm	Leukemia	- EE is more<br>- organic solvent is used in cited literature	*Tima, S., et al 2019*
7.	Nanoparticles	-DOX-Tethered CUR NP<br>-EE: 91%	Liver carcinoma	-Complex formulae of cited NPs	*Rajiu, V., et al 2015*
8.		-PLGA based Cur NPs in acetone	Cervical cancer	-Use of organic solvent in the cited literature	*Zaman, M.S., et al 2016*
9.	Liposomes	-prepared by cationic lipids in chloroform and methanol<br>-PS:208nm<br>-ZP:4.6mV<br>-DL:12%<br>-EE:97%	Skin & breast cancer	- organic solvent used in cited literature<br>- cationic lipids are costly and toxic	*Moku, G., et al 2016*
10.		-prepared with high % of lipids<br>-PS:117nm<br>-ZP:-15.8mV	Synovial sarcoma	-Lower concentration of lipid and surfactants is used in CLEN	*Kloesch, B., et al 2016*
11.		-Curcumin loaded liposomes using chloroform<br>-EE: 81%<br>-PS: 271nm	Asthma	-Aqueous formulae of CLEN versus use of chloroform in cited literature	*Ng, Z.Y., et al 2018*
12.		-POPC liposomes in chloroform<br>-ZP: -32.37<br>-PS: 399nm	Anti-inflammatory effects in restorative dentistry practice	-Use of chloroform and DMSO in the cited literature	*Sinjari, B., et al 2019*

References

Aditya, N.P., Shim, M., Lee, I., Lee, Y.J., Im, M.H., Ko, S., 2013. Curcumin and genistein coloaded nanostructured lipid carriers: in vitro digestion and antiprostate cancer activity. Journal of Agricultural and Food Chemistry 61, 1878-1883.

Alibolandi, M., Hoseini, F., Mohammadi, M., Ramezani, P., Einafshar, E., Taghdisi, S.M., et al. 2018. Curcumin-entrapped MUC-1 aptamer targeted dendrimer-gold hybrid nanostructure as a theranostic system for colon adenocarcinoma. International Journal of Pharmaceutics,11, 1-27.

Bane, C., Joa, M., Parka, Y.H., Kima, J.H., Hana, J.Y., Leea, K.W., Kweon, D.H., Choia, Y.J., 2020. Enhancing the oral bioavailability of curcumin using solid lipid nanoparticles. Food Chemistry 302, 1-10.

Beloqui, A., Memvanga, P.B., Coco, R., Reimondez, T.S., Alhouayek, M., Muccioli, G.G., Alonso, M.J., Csaba, N., Fuente, M., Préat, V., 2016. A comparative study of curcumin-loaded lipid-based nanocarriers in the treatment of inflammatory bowel disease. Colloids and Surfaces b: Biointerfaces 143, 327-335.

Ganesan, P., Kim, B., Ramalaingam, P., Karthivashan, G., Revuri, V., Park, S., Kim, J.S., Ko, Y.T., Choi, D.K., 2019. Antineuroinflammatory activities and neurotoxicological assessment of curcumin loaded solid lipid nanoparticles on LPS-stimulated BV-2 microglia cell models. Molecules 24, 1-11.

Gaur, P.K., Mishra, S., Verma, A., Verma, N., 2016. Ceramide–palmitic acid complex based curcumin solid lipid nanoparticles for transdermal delivery: pharmacokinetic and pharmacodynamic study. Journal of Experimental Nanoscience 11, 38-53.

Ji, H., Tang, J., Li, M., Ren, J., Zheng, N., Wu, L., 2016. Curcumin-loaded solid lipid nanoparticles with Brij78 and TPGS improved in vivo oral bioavailability and in situ intestinal absorption of curcumin. Drug Delivery 23, 459-470.

Ji, S., Lin, X., Yu, E., Dian, C., Yan, X., Li, L., et al. 2018. Curcumin-Loaded Mixed Micelles: Preparation,Characterization, and In Vitro Antitumor Activity. Journal of Nanotechnology, 2018, 1-9.

Jourghanian, P., Ghaffari, S., Ardjmand, M., Haghighat, S., Mohammadnejad, M., 2016. Sustained release curcumin loaded solid lipid nanoparticles. Advanced Pharmaceutical Bulletin 6, 17-21.

Kesharwani, P., Xie L., Banerjee, S., Mao, G., Padhye, S., and Iyer, A. K., 2015. Hyaluronic acid-conjugated polyamidoamine dendrimers for targeted delivery of 3,4-difluorobenzylidene curcumin to CD44 overexpressing pancreatic cancer cells. Colloids and Surfaces B: Biointerfaces, 136, 413-423.

Kloesch, B., Gobe,r L., Loebsch, S., Vcelar, B., Helson, L., Steiner, G., 201). In Vitro Study of a Liposomal Curcumin Formulation (Lipocurc™): Toxicity and Biological Activity in Synovial Fibroblasts and Macrophages. In Vivo, 30 , 413-419.

Madane, R.G., Mahajan, H.S., 2016. Curcumin-loaded nanostructured lipid carriers (NLCs) for nasal administration: design, characterization, and in vivo study. Drug Delivery 23, 1326-1334.

Moku, G., Gulla, S.K., Nimmu, N.V., Khalid, S., and Chaudhuri, A., 2016 . Delivering anti-cancer drugs with endosomal pH-sensitive anti-cancer liposomes. Biomaterial Sciences, 4,627- 638.

Nayak, A.P., Tiyaboonchai, W., Patankar, S., Madhusudhan, B., Souto, E.B., 2010. Curcuminoids-loaded lipid nanoparticles: novel approach towards malaria treatment. Colloids and Surfaces B: Biointerfaces 81, 263-273.

Ng, Z.Y., Wong, J.Y., Panneerselvam, J., Madheswaran, T., Kumar, P., Pillay, V., et al 2018. Assessing the Potential of Liposomes Loaded With Curcumin as a Therapeutic Intervention in Asthma. Colloids Surf B Biointerfaces, 172, 51-59.

Puglia, C., Frasca, G., Musumeci, T., Rizza, L., Puglisi, G., Bonina, F., Chiechio, S., 2012. Curcumin loaded NLC induces histone hypoacetylation in the CNS after intraperitoneal administration in mice. European Journal of Pharmaceutics and Biopharmaceutics 81, 288-293.

Rajiu, V., Balaji, P., Sheena, T.S., Akbarsha, M.A., and Jeganathan, K., 2015. Doxorubicin-Anchored Curcumin Nanoparticles for Multimode Cancer Treatment against Human Liver Carcinoma Cells. Particle Systems Characterization. *32*, 1028–1042.

Righeschi, C., Bergonzi, M.C., Isacchi, B., Bazzicalupi, C., Gratteri, P., Bilia, A.R., 2016. Enhanced curcumin permeability by SLN formulation: the PAMPA approach. LWT-Food Science and Technology 66, 475-483.

Sandhir, R., Yadav, A., Mehrotra, A., Sunkaria, A., Singh, A., Sharma, S., 2014. Curcumin nanoparticles attenuate neurochemical and neurobehavioral deficits in experimental model of Huntington's disease. Neuromolecular Medicine 16, 106-118.
Sarika, P.R., James, N.R., Kumar, P.R.A., Raj, D.K., and Kumary, T.V., 2015. Gum arabic-curcumin conjugate micelles with enhanced loading for curcumin delivery to hepatocarcinoma cells. Carbohydrate Polymers, 134, 167-174.

Shelat, P., Mandowara, V.K., Gupta, D.G., PATEL, S., 2015. Formulation of curcuminoid loaded solid lipid nanoparticles in order to improve oral bioavailability. International Journal of Pharmacy and Pharmaceutical Sciences 7, 278-282.

Sinjari, B., Pizzicannella, J., D'Aurora, M., Zappacosta, R., Gatta, V., Fontana, A., et al 2019. Curcumin/Liposome Nanotechnology as Delivery Platform for Anti-inflammatory Activities via NFkB/ERK/pERK Pathway in Human Dental Pulp Treated With 2-HydroxyEthyl MethAcrylate (HEMA). Frontiers in Physiology, 10: 633.

Sun, J., Bi, C., Chan, H.M., Sun, S., Zhang, Q., Zheng, Y., 2013. Curcumin-loaded solid lipid nanoparticles have prolonged in vitro antitumour activity, cellular uptake and improved in vivo bioavailability. Colloids and Surfaces B: Biointerfaces 111, 367-375.

Sutaria, D., Grandhi, B.K., Thakkar, A., Wang, J., Prabhu, S., 2012. Chemoprevention of pancreatic cancer using solid-lipid nanoparticulate delivery of a novel aspirin, curcumin and sulforaphane drug combination regimen. International Journal of Oncology 41, 2260-2268.

Tima, S., Okonogi, S., Ampasavate, C., Berkland, C., and Anuchapreeda, S., 2019. FLT3-specific curcumin micelles enhance activity of curcumin on FLT3-ITD overexpressing MV4-11 leukemic cells. Drug Development and Industrial Pharmacy, 45, 498-505.

Tripathi, P.K., Gupta, S., Rai, S., Shrivatava, A., Tripathi, S., Singh, S., et al. (2020). Curcumin loaded poly (amidoamine) dendrimer-plamitic acid core-shell nanoparticles as anti-stress therapeutics. Journal of Drug Development and Industrial Pharmacy, 46, 412-426.

Wang, J., Wang, H., Zhu, R., Liu, Q., Fei, J., Wang, S., 2015. Anti-inflammatory activity of curcumin-loaded solid lipid nanoparticles in IL-1β transgenic mice subjected to the lipopolysaccharide-induced sepsis. Biomaterials 53, 475-483.

Wang, W., Chen, T., Xu, H., Ren, B., Cheng, X., Qi, R., Liu, H., Wang, Y., Yan, L., Chen, S., Yang, Q., Chen, C., 2018. Curcumin loaded solid lipid nanoparticles enhanced anticancer efficiency in breast cancer. Molecules 23, 1-13.

Wang, W., Zhu, R., Xie, Q., Li, A., Xiao, Y., Li, K., Liu, H., Cui, D., Chen, Y., Wang, S., 2012. Enhanced bioavailability and efficiency of curcumin for the treatment of asthma by its formulation in solid lipid nanoparticles. International Journal of Nanomedicine 7, 3667.

Zaman, M.S., Chauhan, N., Yallapu, M.M., Gara, R.K., Maher, D.M., Kumari, S., et al 2016. Curcumin Nanoformulation for Cervical Cancer Treatment. Scientific Reports, 6.
